# Cost of diagnosing dementia in a German memory clinic

**DOI:** 10.1186/s13195-017-0290-6

**Published:** 2017-08-22

**Authors:** Bernhard Michalowsky, Steffen Flessa, Johannes Hertel, Olav Goetz, Wolfgang Hoffmann, Stefan Teipel, Ingo Kilimann

**Affiliations:** 1 Deutsches Zentrum fur Neurodegenerative Erkrankungen (DZNE) Standort Rostock/Greifswald, Greifswald, Germany; 2 Department of General Business Administration and Health Care Management, Ernst Moritz Arndt University Greifswald, Greifswald, D-17489 Germany; 3 Department of Psychiatry and Psychotherapy, University Medicine Greifswald, Greifswald, Ellernholzstraße 1-2, D-17487 Germany; 4 Institute for Community Medicine, Section Epidemiology of Health Care and Community Health, University Medicine Greifswald (UMG), Greifswald, Ellernholzstrasse 1-2, D-17487 Germany; 5 Department of Psychosomatic Medicine, University Hospital Rostock, and German Centre for Neurodegenerative Diseases (DZNE), Rostock/Greifswald, Rostock, Gehlsheimer Strasse 20, D-18147 Germany

**Keywords:** Alzheimer’s disease, Dementia, Healthcare economics and organizations, Diagnoses, Healthcare cost

## Abstract

**Background:**

Little is known about diagnostic work-ups or the costs of diagnosing dementia in specialized care. Here, we analyzed the costs of diagnosing dementia according to specific dementia disorders.

**Methods:**

A prospective descriptive design was used to analyze the cost of diagnosing dementia for 120 patients with suspected dementia at a German memory clinic. The duration of clinical consultations and use of technical procedures were recorded by the memory clinic staff. To detect cost drivers, a multiple linear regression model was used.

**Results:**

Of patients with suspected dementia, 44% were diagnosed with dementia. The total cost per patient and diagnostic process amounted to 501 € across all patients and 659 € for patients who were diagnosed with dementia. The costs varied between 649 € for patients with Alzheimer’s disease, 662 € for patients with vascular or mixed dementia, and 705 € for patients with unspecific dementia. A final diagnosis of dementia was the only factor that was significantly associated with the diagnostic cost (*b* = 356, CI^–^ 182, CI^+^ 531, *p* = 0.001).

**Conclusion:**

The high range of costs reflects differences in diagnostic demands depending on the etiology of dementia. This variation needs to be transferred into reimbursement. Further studies are needed to assess the influence of the type of cognitive impairment and of the setting on diagnostic costs.

**Electronic supplementary material:**

The online version of this article (doi:10.1186/s13195-017-0290-6) contains supplementary material, which is available to authorized users.

## Background

The syndrome of dementia is characterized by a loss of memory and other mental abilities that are severe enough to interfere with daily life [1]. Most often, dementia is caused by a chronic and progressive neurodegenerative disease, usually Alzheimer’s disease. Worldwide, there are more than 46.8 million persons living with dementia. This number is expected to double every 20 years, reaching 74.7 million in 2030 and 131.5 million in 2050 [2–4]. In Germany, the current number of persons with dementia is estimated to be over 1.6 million, with an annual incidence of over 300,000 new cases [5]. From an economic point of view, dementia is the main cause of long-term institutional care in the older population and is therefore associated with substantial healthcare costs [6]. Specifically, the total worldwide cost of dementia was estimated at 784 billion € (US$ 818 billion; 1 € = US$ 1.043, exchange rate as of 16 December 2016) in 2016. Thus, dementia is one of the most expensive diseases in old age [2, 7].

Persons with dementia require a timely diagnosis as a basis for adequate and cost-effective drug and nondrug treatments to delay the progression of the disease and diminish increasing healthcare costs [8, 9]. Diagnosing dementia involves determining the presence of dementia as well as nosological diagnosis of the specific causes of the syndrome.

The criteria for diagnosing Alzheimer’s disease, the most frequent cause of dementia in older people, can serve as a blueprint for dementia diagnosis procedures. In agreement with international guidelines [10–12], the national guidelines on dementia care of the German Association for Psychiatry, Psychotherapy and Psychosomatics and the German Association for Neurology [13] recommend using the clinical history, medical and neurological examinations, and assessments of cognitive functions (e.g., Mini-Mental State Examination (MMSE) [14, 15], DemTect [16], Clock Drawing Test [17]) in the first step. If the syndrome of dementia or mild cognitive impairment (MCI) is detected, further investigations include blood sampling and cranial computer tomography (CT) or magnetic resonance imaging (MRI) on a regular basis. Under certain circumstances, biomarker-based diagnosis is recommended, including neurodestruction markers from cerebrospinal fluid puncture (CSF) and metabolic and molecular markers from positron emission tomography (PET). Complete adherence to these recommendations will typically only be possible in specialized care settings, such as in memory clinics. Primary care guidelines are oriented around these recommendations and allow the diagnosis of dementia diseases in primary care as well (e.g., the guidelines of the German College of General Practitioners and Family Physicians – DEGAM [18]), but they are rarely implemented in clinical routine [19–21]. Approximately 50–80% of persons with dementia do not receive a dementia diagnosis [19–21], and between 45 and 55% of persons with a dementia diagnosis receive a diagnosis of unspecific dementia in primary care [22, 23].

Only two studies have so far evaluated the diagnostic work-ups and cost of diagnosing dementia in Sweden [24, 25]. The cost per diagnosed patient has been estimated to be between 477 € (US$ 497) at the primary care level and 1115 € (US$ 1163) at the specialist level. Wimo et al. [24] further identified age and cognitive impairment as crucial cost drivers. However, little is known about cost differences in diagnosing dementia referring to specific dementia disorders, such as Alzheimer’s diseases or cerebrovascular disease. The costs of dementia diagnosis in different settings and related to different underlying causes of dementia are important for assessing the feasibility of a more rigorous implementation of diagnostic procedures in routine care, including primary care.

### Aims of the study

The objectives of this study were to analyze the cost of diagnosing dementia in patients with suspected dementia at a German memory clinic in specialized care, to evaluate the differences in the cost of diagnosing dementia in relation to different dementia disorders, and to determine sociodemographic and clinical factors associated with the cost of diagnosing dementia.

## Methods

### Study design and setting

A prospective descriptive design was conducted to analyze the cost of diagnosing dementia for patients with suspected dementia diagnosed at a German memory clinic in specialized care. The diagnostic work-ups of the memory clinic followed the guidelines of the German Society of Neurology (DGN) and the German Society of Psychiatry, Psychotherapy and Neurology (DGGPN), integrating biomarkers into the diagnostic procedure on a routine basis [26]. Patients with subjective cognitive decline were not included in a biomarker-based diagnostic work-up as the validity of biomarkers in this patient group is still low. Patients with suspected dementia passed through the entire diagnostic process from June 2015 to June 2016. The diagnostic process starts with the patient’s administrative registration and ends with the determination of the final diagnosis recorded in the physician’s letter. We divided the diagnostic process into clinical consultations with the staff of the memory clinic and the utilization of technical procedures. Clinical consultations include the patient’s administrative admission, anamnesis, physical examination, neuropsychological examination, test evaluation, diagnosis conference, discussion of results and preparation (voice recording by neurologist/psychiatrist), and writing of a physician letter (audiotyping by team assistant). Clinical consultations were conducted by team assistants, neurologists, psychiatrists, psychologists, and psychological assistants. Technical procedures include all procedures, such as MRI, CT, CSF, and PET, as well as blood tests. In this analysis we focused on patients’ first consultations. A diagnosis was stated after all of the results were gathered in the diagnosis conference. Follow-up examinations and a possible change in the diagnosis over time were not part of this study. Additional file 1 demonstrates the entire diagnostic process. The trial has been discussed in detail and has been approved by the Ethical Committee and the Workers’ Council of the Medical Faculty of the University Rostock, Germany (registry number A 2011-0046).

### Study sample

Overall, 124 patients provided written informed consent (IC) to participate in the trial. There were four patients excluded from the analysis because of missing data concerning patients’ cognitive impairment. Therefore, the final analysis was based on a total of 120 patients.

### Assessment of the diagnostic procedure

For the personnel-related and time-related clinical consultations, the duration of each consultation was independently self-recorded by the staff of the memory clinic. The number of staff involved in each consultation and their profession were also self-recorded. Self-recorded measurements have proven to be as exact as stopwatch measurements of medical treatment and diagnostic procedures in nonemergency clinical settings [27]. Utilization of technical procedures was assessed from patient medical records. Imaging procedures and blood tests that were conducted previously at other ambulatory or inpatient facilities were also documented using patients’ medical records. To calculate the total cost of diagnosing dementia, technical procedures that were conducted previously at other medical facilities and technical procedures conducted at the memory clinic were summed.

### Sociodemographic and clinical variables

To analyze associations with the cost of diagnosing dementia, the following sociodemographic and clinical variables were included: age, sex, cognitive impairment, comorbidity, number of drugs taken, and final ICD-10 diagnosis (International Statistical Classification of Diseases and Related Health Problems) [28]. The severity of cognitive impairment was assessed using the MMSE [14, 15]. Based on the suggestions for dementia severity grading in Alzheimer’s disease according to the German S-3 guideline, we categorized participants into one of four groups of cognitive impairment: without (MMSE score ≥ 27), mild (MMSE score 20–26), moderate (MMSE score 10–19), and severe (MMSE score 0–9) [13]. Because of a small number of patients with moderate to severe cognitive impairment, these two categories were collapsed into one. The final ICD-10 diagnoses were obtained from patients’ medical records and were based on the full four-digit general scheme (e.g., F00.0, F00.1, F01.1) or on a three-digit scheme representing a group of diagnoses (e.g., F03). In this analysis, patients diagnosed with dementia were defined as patients who received the following ICD-10 diagnoses: F00.0 and F00.1 for Alzheimer’s disease dementia; F01.1 for vascular dementia; F00.2 for mixed dementia; F02.0 for fronto-temporal dementia; and F03 for unspecific dementia. Because of a small number of patients with vascular and mixed dementia, both specific disorders were pooled. We furthermore differentiated between patients with MCI (F06.7), patients with subjective cognitive complaints due to other conditions (F33.1, F34.1, F41.2, F31.1, and F60.3), and patients with other disorders or patients with no evidence of cognitive impairment and no subjective memory complaints. To assess patients’ comorbidities and medications, the physicians asked each patient, and if possible their relatives, for their health and medical history as well as their regularly taken drugs. The total number of previous illnesses and surgeries as well as the number of regularly taken drugs were counted as simple comorbidity and medication scores, respectively.

### Cost analysis

A bottom-up design was used to assess the average costs per diagnostic procedure in patients with suspected dementia, in finally diagnosed patients with specific dementia disorders, and in true dementia cases. The cost for a true dementia diagnosis includes the costs of all of the conducted investigations divided by the number of final dementia diagnoses. All of the costs were assessed using the unit costs for the different components of the diagnostic process. For time-related clinical consultations, we used the average per-minute nationwide gross wages of employees in Germany, which includes nonwage labor costs [29–31]. We furthermore estimated the overhead cost for clinical consultations to be 20%. The average loss of working time due to leave or sickness was estimated to be 15%. Technical procedures were monetized using the pricelist for the clinical procedures of the German Hospital Federation [32]. These costs refer to the full cost, including the overhead costs (administration, housing, staff, etc.), material costs, and cost of medical services. The costs were calculated in euros (€) at 2016 price levels (1 € = US$1.043, exchange rate as of 16 December 2016). When prices were not available for the year 2016, the prices were inflated by the individual inflation rate of recent years [33]. Additional file 2 demonstrates the methods of the cost calculation and unit costs used.

### Statistical analysis

We used descriptive statistics to analyze the sociodemographic and clinical data of the patients, and technical procedures were utilized to identify biomarkers. Differences in means (proportions) were evaluated using *t* tests (Fischer’s exact test). To handle missing values of the time-related clinical consultations, we used multiple imputations via chained equations (MICE) with the imputation model being an ordinary least squares regression containing the technical procedures used, patients’ age, MMSE, comorbidity, number of drugs taken, and final ICD-10 diagnosis. MICE has emerged as a principled flexible method of dealing with missing data and is particularly useful for data where the parametric assumption made in joint modeling procedures may not be appropriate [34, 35]. Thus, MICE was used to estimate appropriate and conservative confidence intervals (CIs) for the costs. In total, 20 imputed datasets were generated by MICE.

Additional file 3 demonstrates the number of missing values for each time-related clinical consultation. To calculate minimum and maximum values, we used the mean imputation.

To analyze the association between the cost of diagnosing dementia and further sociodemographic and clinical data, a multiple linear regression model was conducted averaging the models over the 20 MICE-generated datasets [34, 35]. Age, sex, MMSE, comorbidity, number of drugs taken, and final ICD-10 diagnoses were included as covariates to minimize confounding. Statistical analyses were conducted using the software STATA/SE version 13.0 [36].

## Results

### Patients’ sociodemographic and clinical characteristics

Table 1 presents the characteristics of the total sample.

**Table 1:** Sociodemographic and clinical characteristics of the study population at a memory clinic in primary care

	Total sample (*n* = 120)
Age	
Mean (SD)	72.8 (9.5)
Range	49–91
Sex, *n* (%)	
Female	72 (60.0)
MMSE	
Mean (SD)	25.3 (5.5)
Range	0–30
Severity of cognitive impairment^a^, *n* (%)	
No indication	71 (59.2)
Mild	35 (29.2)
Moderate to severe	14 (11.6)
Final ICD-10 diagnosis after diagnostic procedure, *n* (%)	
Dementia^b^	53 (44.2)
Alzheimer’s diseases (F00.0 and F00.1)	30 (24.2)
Vascular dementia (F01.1)	16 (13.3)
Mixed dementia (F00.2)	2 (1.7)
Unspecific dementia (F02.0 and F03)	5 (4.0)
MCI (F06.7)	28 (22.6)
Other conditions	39 (32.5)
Subjective disorders (F33.1, F34.1, F41.2, F31.1, and F60.3)	33 (27.5)
No hint for cognitive disorders	3 (2.5)
Other	3 (2.5)
Number of existing illnesses and previous surgeries	
Mean (SD)	3.9 (2.8)
Range	0–16
Number of drugs regularly taken	
Mean (SD)	5.0 (3.5)
Range	0–16

^a^According to MMSE

^b^Referring to the following ICD-10 diagnoses: F00.0, F00.1, F01.1, F00.2, F02.0, and F03

*MMSE* Mini-Mental State Examination (range 0–30, higher score indicates better cognitive function), *ICD* International Statistical Classification of Diseases and Related Health Problems, *SD* standard deviation

### Diagnosing dementia diseases and other conditions

After the entire diagnostic procedure, 44% of patients received a dementia diagnosis, 23% an MCI diagnosis, and 33% another conditions diagnosis or had no indication of cognitive impairments or complaints. The most frequent diagnoses were Alzheimer’s disease, with 30% (F00.0, F00.1), and subjective memory complaints, with 33% (F33.1, F34.1, F41.2, F31.1, F60.3); 4% of patients received the diagnosis “unspecific dementia”. ICD-10 diagnoses are reported in Table 1.

### Utilization of diagnostic procedures to identify biomarkers

The utilization of diagnostic procedures is presented in Table 2. MRI was the most frequently used technical procedure (59%), followed by a blood test (37%). CSF, CT, and PET were utilized less frequently (18%, 17%, and 6%, respectively). Patients diagnosed with dementia received blood tests (51% vs 25%), MRI (72% vs 49), CT (26% vs 9%), and PET (11% vs 5%) significantly more often.

**Table 2:** Utilization of diagnostic procedures to identify biomarkers and average costs of the diagnostic process for the total sample and depending on the subsequently received dementia diagnosis

	Total sample (*n* = 120)			Patients who were finally diagnosed with dementia^a^ (*n* = 53)			Patients who were finally not diagnosed with dementia^b^ (*n* = 67)			*p* value^c^
	*n* (%)			*n* (%)			*n* (%)			
Utilization of procedures										
Blood test	44 (36.7)			27 (50.9)			17 (25.4)			0.005
MRI	71 (59.2)			38 (71.7)			33 (49.3)			0.016
CT	20 (16.7)			14 (26.4)			6 (8.9)			0.014
CSF	22 (18.3)			11 (20.8)			11 (16.4)			0.637
PET	7^d^ (5.8)			4^d^ (7.5)			3 (4.5)			0.698
Cost of diagnosis	Mean	95CI^–^	95CI^+^	Mean	95CI^–^	95CI^+^	Mean	95CI^–^	95CI^+^	
Time-related processes (€)	110	105	115	121	114	128	103	96	109	0.002
Diagnostic procedures (€)	391	320	460	538	421	655	274	199	348	0.001
Total diagnostic process (€)	501	430	573	659	540	778	376	301	451	0.001

Differences in means were evaluated using two-tailed *t* tests referring to patients diagnosed with dementia and without dementia diagnosis

*MRI* magnetic resonance imaging, *CT* computer tomography, *CSF* cerebrospinal fluid puncture, *PET* positron emission tomography, *95CI* 95% confidence interval, *ICD* International Statistical Classification of Diseases and Related Health Problems

^a^Referring to the following ICD-10 diagnoses: F00.0, F00.1, F01.1, F00.2, F02.0, and F03

^b^Referring to the following ICD-10 diagnoses: F06.7, F33.1, F34.1, F41.2, F31.1, and F60.3, and other conditions or no hint for cognitive impairment

^c^Differences in proportions evaluated using Fischer’s exact test, differences in means evaluated using *t* tests

^d^One patient received florbetaben PET, all others fluorodeoxyglucose PET

### Costs of diagnosing dementia

**Fig. 1:**
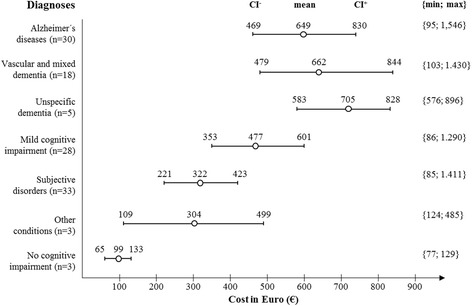
Cost of the diagnostic process at a memory clinic in primary care referring to different final diagnoses (mean values, lower and upper CIs, as well as minimum and maximum values). Minimum and maximum values calculated using the mean imputation; CIs and mean values calculated using MICE. Alzheimer’s disease, F00.0 and F00.1; vascular and mixed dementia, F01.1 and F00.2; unspecific dementia, F02.0 and F03; MCI, F06.7; subjective disorders, F33.1, F34.1, F41.2, F31.1, and F60.3. *CI* confidence interval

The total cost per patient with suspected dementia per complete diagnostic process was 501 € (US$523), including 110 € (US$115) for the clinical consultations and 391 € (US$408) for technical procedures. For those who received a dementia diagnosis, the average diagnostic cost was significantly higher (659 €/US$687) compared to those who received a diagnosis of MCI or subjective memory complaints or other conditions (376 €/US$392). The most cost-intensive diagnostic process was used in patients who received the diagnosis unspecific dementia after a comprehensive examination (705 €/US$735). The cost of diagnosing Alzheimer’s diseases or vascular/mixed dementia was valued at 649 € (US$676) and 662 € (US$648), respectively. According to the severity of the cognitive impairment, the cost of the entire diagnostic process was higher in patients with MCI (653 €/US$681) compared to those with either no hint of it (434 €/US$452) or with moderate to severe cognitive impairment (543 €/US$566). Costs of diagnosing dementia and other conditions are shown in Table 2 and Fig. 1.

**Fig. 2:**
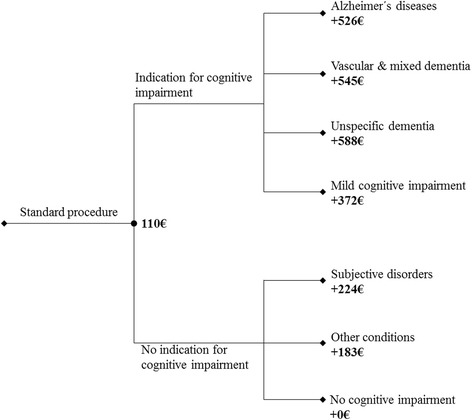
Cost of standard clinical consultations and excess costs including the cost of technical procedures and further comprehensive neuropsychological assessments. Alzheimer’s disease, F00.0 and F00.1; vascular and mixed dementia, F01.1 and F00.2; unspecific dementia, F02.0 and F03; MCI, F06.7; subjective disorders, F33.1, F34.1, F41.2, F31.1, and F60.3

The cost of the standard procedures, which includes clinical consultations but excludes the specific technical procedures used as well as further comprehensive neuropsychological assessments, was 110 € (US$104.3). To clarify cognitive impairment, the excess costs were between 372 € for the detection of MCI and 588 € for diagnosing unspecific dementia after a comprehensive examination. The total cost of the entire diagnostic process for all 120 patients was valued at 60,120 € (US$62,705), meaning that the detection of one true dementia patient (*n* = 53) in this sample was associated with a cost of 1134 € (US$1183). Thus, the cost for detecting nondementia cases was valued at 897 €. Figure 2 presents the total cost of the standard procedure that all patients had to pass through as well as the excess cost for the detection of specific dementia disorders.

### Association between diagnostic cost and sociodemographic and clinical variables

Table 3 presents the results of the linear mixed regression model. Adjusted for age, sex, comorbidity, cognitive impairment, and number of drugs taken, a dementia diagnosis (referring to the ICD-10 diagnoses F00–F03) was the only factor positively associated with the cost of the diagnostic process (*b* = 356, CI^–^ 181, CI^+^ 531, *p* = 0.001).

**Table 3:** Multivariate regression model of cost drivers

	Total cost of diagnostic procedure		
	*b* (SE)	95% CI	
Age	0.1 (4.2)	– 8.2	8.4
Sex (reference: female)	– 71.3 (70.8)	– 211.5	69.0
Severity of dementia (MMSE)^a^	11.3 (7.9)	– 4.3	27.0
Final diagnosis (reference: dementia^b^)	356.3 (88.1)**	181.8	530.8
Number of existing illnesses and previous surgeries	– 4.8 (15.3)	– 35.1	25.5
Number of drugs regularly taken	– 4.1 (12.5)	– 28.8	20.6
Intercept	133.3 (392.8)*	– 645.1	911.8

Linear mixed model: 120 observations, *p* = 0.003

*p < 0.01, **p < 0.001 (statistically significant)

^a^According to Mini-Mental State Examination (*MMSE*): values reverse coded, range 0–30, higher scores indicate better cognitive function

^b^Referring to the following ICD-10 diagnoses: F00.0, F00.1, F01.1, F00.2, F02.0, and F03

b observed coefficient, *SE* standard error, *CI* confidence interval, *ICD* International Statistical Classification of Diseases and Related Health Problems

## Discussion

This study demonstrates the costs of diagnosing different dementia disorders for patients with suspected dementia in a specialized memory clinic on the basis of assessed time-related clinical consultations and the specific technical procedures used to identify relevant biomarkers. The total cost per patient and process was 501 €, and was 659 € for patients who were diagnosed with dementia. The cost of diagnosing different dementia disorders varied between 649 € for patients diagnosed with Alzheimer’s disease and 705 € for patients diagnosed with unspecific dementia. The cost of a true dementia case (nondementia case) was 1134 € (897 €).

These costs compare with the slightly higher costs reported in two previous studies that amounted to 1334 € and 1298 €, respectively, for diagnosing a true dementia case and 1115 € per patient with suspected dementia at a specialized care level [24, 25]. Jedenius et al. [25] evaluated the total costs associated with diagnosing dementia diseases from the beginning of the diagnostic process to the time when a dementia diagnosis was established or rejected using a prospective time-related and resource-related study, which is comparable to the methods used in our analysis. However, only 40 patients with suspected dementia at the age of 83 were included. The sample of Wimo et al. [24] was at a comparable mean age of 82. Thus, these two previous samples were older than those in our analysis (mean age 73). Because of the exponential increase of dementia prevalence with age, the inclusion of an older sample with suspected dementia will likely lead to a higher rate of finally diagnosed dementia patients. In the study by Jedenius et al. [25], two-thirds of patients were diagnosed with dementia diseases at the end of the diagnostic process. This rate is higher compared with those reported in another longitudinal trial [37]. This trial reported that, on average, 50% of patients with suspected dementia had a confirmed diagnosis, which is comparable to the 44% in our sample. This lower rate of dementia cases should lead to a higher cost for one true dementia case. However, the cost of diagnosing dementia in our study was favorable compared to previous studies. The major reason for the lower evaluated costs could be the variability in the technical procedures used and in the cost of diagnostic procedures between different countries. The unit costs for the staff involved in the diagnostic processes as well as for the diagnostic procedures used, such as CT, CSF, and MRI, were slightly higher in Sweden [24]. For example, the reimbursement rate for a CSF (CT) was 130 € (217 €) in specialized care in Sweden, but 118 € (172 €) in Germany, if the purchasing-power parity in both countries remains unconsidered. Furthermore, based on a subsample of only 10 patients, Jedenius et al. [25] estimated the costs for diagnosing dementia by adding the entire diagnostic cost at the primary care level (general practitioner based) to the cost of diagnosing dementia at the specialist level. In this analysis, primary care consultations are not taken into account and primary care technical procedures conducted previously are solely included. These could be the reasons for the demonstrated higher costs compared to the study of Jedenius et al. [25]. However, it seems that the demonstrated costs are estimated under uncertainty, and thus further research is needed to confirm these estimates with larger samples in different settings.

The wide variation of cost associated with different dementia disorders is related to differences in the complexity of diagnostic processes as well as to higher frequencies of imaging or biomarker-based diagnostic procedures. The diagnosis of “unspecific dementia” was given in five cases and was associated with the highest costs. Compared to patients diagnosed with Alzheimer’s diseases, vascular dementia, or mixed dementia, the unspecific dementia diagnosis after a comprehensive examination was associated with longer process times, higher utilization of diagnostic procedures, and enhanced neuropsychiatric tests. This was due to the fact that those with dementia disease presented with more atypical features compared with the other patients.

In Germany, reimbursement for a diagnostic process in a memory clinic is neither standardized nor adjusted for different dementia diagnoses and their associated costs. The financing of memory clinics could therefore differ tremendously. First of all, “memory clinic” is not a protected term, leading to several possibilities of institutional connections. Thus, each memory clinic has an individual basis for the reimbursement. In most cases, reimbursement is based on flat-rate payments or special agreements with health insurers, and the financial means provided by those insurers can be insufficient to fully cover the cost of the diagnostic process of each patient. Therefore, most clinics use additional funds for compensation, such as donations [38]. However, reimbursement is not based on procedures that are necessary to reach a final diagnosis. It could be possible that this represents an incentive toward fewer diagnostic procedures and declaring a diagnosis with greater remaining uncertainty, especially for untreatable forms of dementia. Paying by the final diagnosis would seem to create an incentive to identify specific dementia diseases. This may be informed by the sequence of diagnostics that are typically performed. However, it may be open to manipulation in the future. Thus, designing a reimbursement system that aligns the incentives of physicians, patients, and the public payer could be very difficult. Even though sufficient funding by health insurance would enable annual planning of diagnostic processes and sustainable reimbursement, memory clinics are actually not standardized according to their medical services or their connection to hospital structures. The development of homogeneous structures could help to establish memory clinics more often and to initiate sustainable standardized funding. The evaluation of cost differences when diagnosing dementia diseases is very important to standardizing a reimbursement system for memory clinics.

However, the currently unstandardized financing structure represents a risk for diagnostic facilities, such as memory clinics. Diagnostic processes for atypical cases are by far the most expensive in a specialized memory clinic, even if only a small proportion of the total sample (4%) is diagnosed with unspecific dementia. In primary care, between 45 and 55% of persons with dementia receive an unspecific dementia diagnosis by general practitioners [22, 23]. However, Wucherer et al. [39] revealed that a dementia diagnosis is not a prerequisite for a guideline-based medication treatment in primary care: 38% of cognitively impaired GP patients without dementia diagnosis are treated with anti-dementia drugs. Thus, it is unclear why there are many undiagnosed and unspecific cases in GP practices. Given that cases with unspecific dementia in memory clinics are few (4% in this analysis) and complex (highest average cost in this analysis), it can be assumed that cases of unspecific dementia in primary care are probably clear but seemingly not fully clarified with the risk of inadequate treatment. However, this is neither clear nor established.

The shift of diagnostic criteria from phenomenological to cost-intensive biomarker-based procedures could increase the number of specific dementia disorders and reduce cases with unspecified dementia, leading to adequate treatment. Therefore, reimbursement for such procedures has the potential to improve dementia treatment and reduce overall dementia costs per person, for example, due to an initiated anti-dementia drug treatment resulting in delayed progression and finally institutionalization [40]. The many non-clarified cases in primary care are relevant to determining excess costs for dementia diagnosis in primary care.

According to different cost drivers of diagnostic procedures in dementia, Wimo et al. [24] revealed that in newly diagnosed dementia patients, their age and cognitive impairment were significantly associated with a higher cost of diagnosing dementia diseases. Their retrospective analysis includes a sample of diagnosed patients with dementia and no patients with suspected dementia. For patients with suspected dementia, we found that the final dementia diagnosis (dichotomous: yes, no) was the only factor associated with a higher cost of diagnosing dementia diseases after adjusting for age, sex, comorbidity, medication, and cognitive impairment. If the categorization of the MMSE was included and the final dementia diagnosis excluded from the multivariate model, MMSE score 20–26 was significantly associated with a higher cost for the entire diagnostic process (*b* = 214, CI^–^ 46, CI^+^ 382, *p* = 0.01). Thus, this observation demonstrates the nonlinear correlation between the costs of diagnosing dementia and patients’ cognitive impairment, leading to a peak in patients with MCI. This finding, however, seems plausible and comparable to the finding of a higher cost for patients with unspecific dementia. Whereas fewer diagnostic processes are needed after detecting severe or no cognitive impairment, mild cognitive cases have to be clarified using further comprehensive neuropsychological assessments and technical procedures, resulting in higher cost for the total diagnostic process.

Furthermore, we found evidence for a negative association between diagnostic costs and patients’ age (*b* = – 17, CI^–^ – 34, CI^+^ 0, *p* = 0.054) if only newly diagnosed patients were included in the multivariate model. This is comparable with the findings of Wimo et al. [24]. A possible explanation could be that, in older patients, a decision must be made between the strain and stress imposed by the diagnostic procedure and a precise diagnosis. Because of the possibility of drug-related problems in older age, it is therefore possible that these patients were less likely to be treated with anti-dementia drugs, meaning that the distinction between Alzheimer’s disease and other dementia disorders may be regarded as less important [24]. In addition, older patients are more comorbid, which often results in polypharmacy. Older patients are more likely to have medical conditions that lead to an adapted diagnostic work-up; for example, omitting CSF due to anticoagulation or CT instead of MRI due to the existence of an implanted pacemaker. Furthermore, the association between age and cost could occur due to the possibility that younger patients, who are mostly less cognitively impaired, receive more tests and more diagnostic procedures to clarify their deficits in cognition. However, this study indicated that sociodemographic and clinical factors do not have any impact on total diagnostic expenditures in a sample of patients with suspected dementia.

The overall cost of diagnosing the total sample of 120 patients was, on average, 60,000 €, meaning that the cost of a true dementia case was 1134 €. First, it is important to note that diagnosing dementia is not the only function of a memory clinic; its tasks also include the diagnosis of different neuropsychiatric disorders. However, if we assume that approximately one-third of the 300,000 new incident dementia cases in Germany are being referred to specialized memory clinics [5], the national diagnostic cost would be more than 113 million €. However, Germany spent over 10.5 billion € annually for persons with dementia from a payer perspective [41]. Thus, the cost of diagnosing dementia disease represents only a small proportion of 1% at the current rate of referral. However, a timely diagnosis allows prompt initiation of pharmacological and nonpharmacological interventions and prevents inappropriate treatment of patients with false-positive diagnoses [42, 43]. These opportunities could lead to a reduction in healthcare costs, especially due to a delayed need for care and institutionalization [44–46]. Thus, this small percentage of diagnostic costs among total expenditures on dementia diseases can result in a substantial reduction of lifetime patient costs, exceeding the cost of diagnosing dementia. Lee et al. [42] underlined that the cost-effectiveness of biomarker analysis depends critically on the prevalence of Alzheimer’s disease in the tested population. Specifically, patients with suspected dementia referred to memory clinics have a higher pretest prevalence of Alzheimer’s disease (exceeding 15%) than patients with memory complaints in, for example, GP practices. This fact leads to potential cost savings and thus to cost-effectiveness [42]. However, currently there is no curative treatment available for dementia diseases. For the upcoming disease-modifying therapies, biomarker-based diagnosis will be even more relevant because these new therapies will be very expensive and only effective in a small group of patients with specific clinical and biomarker characteristics. Cost-effectiveness of these therapies will be strongly related to the reliability and validity of diagnosis. The demonstrated results provide prerequisites for such analyses, especially the diagnostic costs identified for different dementia disorders.

Our study has some limitations. First, our data were derived from only one memory clinic in Germany. Furthermore, there were some missing values for each assessed clinical consultation. For two processes (test evaluation and preparation of the physician letter), 40–57% of the duration data were missing. To handle these missing values, we used univariate imputation by linear regression. This method has emerged as a principle method for dealing with missing data and is particularly useful for large imputation procedures. According to the estimated costs, the clinical consultations conducted by staff of the memory clinic represent only a small proportion of the total cost compared to the costs of the technical diagnostic procedures, such as imaging or blood and CSF testing. Therefore, any discrepancies in cost due to missing values for time-related clinical consultations should not be too large. In addition, test evaluation and preparing the physician letter are relatively uniform procedures so that the margin of possibly induced errors appears to be small. Second, the sample size was low, meaning that the demonstrated costs of diagnosing dementia are not representative for the entire population of patients with suspected dementia in Germany, especially for the demonstrated cost of diagnosing unspecific dementia (*n* = 5). However, the proportion of finally diagnosed patients with suspected dementia is comparable to that in other longitudinal studies. Therefore, it seems that the demonstrated costs of diagnosing dementia are representative for newly diagnosed patients with suspected dementia in specialized care.

## Conclusion

The cost of diagnosing different dementia disorders varied between 649 € for patients diagnosed with Alzheimer’s disease and 705 € for patients diagnosed with unspecific dementia, representing solely a small percentage of diagnostic costs among total expenditures on dementia diseases. However, the currently unstandardized financing structures of memory clinics differ tremendously and thus represent a risk for such diagnostic facilities. Designing a reimbursement system that aligns the incentives of physicians, patients, and the public payer could be very difficult but is of vital importance to expand the use of biomarkers in the diagnostic procedure on a routine base. However, evidence concerning the cost for diagnosing specific dementia diseases in different settings is actually missing. It would be in patients’ interest to support an adequate and comprehensive diagnostic process creating the foundation for adequate treatment, without leading to economic inefficiency that could jeopardize the sustainability of a memory clinic or practices in primary care. Therefore, more studies are needed to gather more information regarding diagnostic processes in dementia and regarding the cost of diagnosing dementia. Therefore, it is of high interest to assess the cost of diagnosing dementia in a larger multicenter sample of patients with suspected dementia, especially in different countries, healthcare systems, and healthcare settings.

## Additional files

Additional file 1: Figure showing the process of diagnosing dementia diseases in a German memory clinic. *CSF* cerebrospinal fluid puncture, *CT* computer tomography, *MRI* magnetic resonance imaging, *PET* positron emission tomography. (TIF 186 kb)

Additional file 2: Table presenting methods for monetary valuation of the diagnostic processes and utilized procedures for the identification of biomarkers. ^‡^ Cost for overhead (20%) and absent days due to holiday and sickness (16%) were included. ^‡‡^ Refer to full costs including material costs and cost of medical services. ^†^ Includes the following tests: hemoglobin, hematocrit, erythrocytes, leukocytes, thrombocytes, folic acid and/or vitamin B12, glutamate oxalacetate transaminase, aspartate aminotransferase, glutamate pyruvate transaminase, alanine aminotransferase, gamma-glutamyltranspeptidase, gamma-glutamyltransferase, thyroid stimulating hormone, cholesterol, high-density lipoprotein cholesterol, low-density lipoprotein cholesterol, creatine kinase. ^1^ Grade: “E6”/“3 years of vocational training”; experience level “2” (in the 4th year). ^2^ Grade: “Ä2”/“medical specialist”; experience level “2” (in the 4th year). ^3^ Grade: “E13”/“University degree”; experience level “2” (in the 4th year). ^4^ Grade: “E6”/“3 years of vocational training”; experience level “2” (in the 4th year) (DOCX 16 kb)

Additional file 3: Table presenting a description of the time-related processes as well as the number and percentage of missing values that were imputed for each step in diagnosing dementia. ^‡^ Voice recording conducted by neurologist/psychiatrists. ^‡‡^ Written description of the voice recording. *SD* standard deviation (DOCX 14 kb)

## Abbreviations

*b*: Observed coefficient
CI: Confidence interval
CSF: Cerebrospinal fluid puncture
CT: Computer tomography
IC: Informed consent
ICD: International Statistical Classification of Diseases and Related Health Problems
MMSE: Mini-Mental State Examination
MRI: Magnetic resonance imaging
PET: Positron emission tomography
SD: Standard deviation
