# Supplementary figures and images for: Cost of diagnosing dementia in a German memory clinic
Source: Alzheimers Res Ther. 2017 Aug 22;9:65. doi: 10.1186/s13195-017-0290-6 (PMC5568303; doi:10.1186/s13195-017-0290-6)

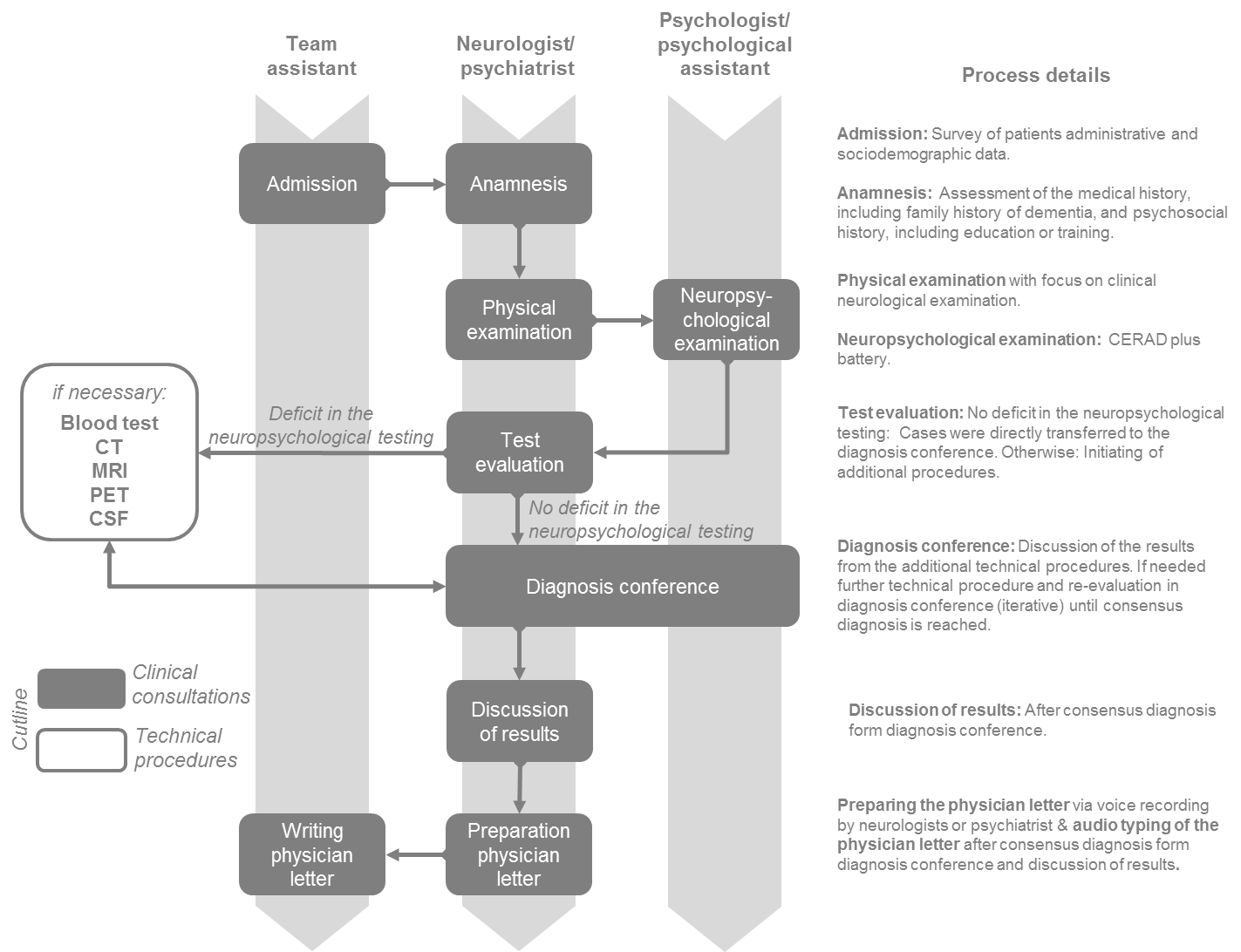

Supplement: Supplementary file 1 — Figure showing the process of diagnosing dementia diseases in a German memory clinic. CSF cerebrospinal fluid puncture, CT computer tomography, MRI magnetic resonance imaging, PET positron emission tomography. (TIF 186 kb) [file 13195_2017_290_MOESM1_ESM.tif]
