# Supplementary material for: Cost of diagnosing dementia in a German memory clinic
Source: Alzheimers Res Ther. 2017 Aug 22;9:65. doi: 10.1186/s13195-017-0290-6 (PMC5568303; doi:10.1186/s13195-017-0290-6)
Supplement: Supplementary file 2 — Table presenting methods for monetary valuation of the diagnostic processes and utilized procedures for the identification of biomarkers. ‡ Cost for overhead (20%) and absent days due to holiday and sickness (16%) were included. ‡‡ Refer to full costs including material costs and cost of medical services. † Includes the following tests: hemoglobin, hematocrit, erythrocytes, leukocytes, thrombocytes, folic acid and/or vitamin B12, glutamate oxalacetate transaminase, aspartate aminotransferase, glutamate pyruvate transaminase, alanine aminotransferase, gamma-glutamyltranspeptidase, gamma-glutamyltransferase, thyroid stimulating hormone, cholesterol, high-density lipoprotein cholesterol, low-density lipoprotein cholesterol, creatine kinase. 1 Grade: “E6”/“3 years of vocational training”; experience level “2” (in the 4th year). 2 Grade: “Ä2”/“medical specialist”; experience level “2” (in the 4th year). 3 Grade: “E13”/“University degree”; experience level “2” (in the 4th year). 4 Grade: “E6”/“3 years of vocational training”; experience level “2” (in the 4th year) (DOCX 16 kb) [file 13195_2017_290_MOESM2_ESM.docx]

Supplementary Table 1: Methods for monetary valuation of the diagnostic processes and utilized procedures for the identification of biomarkers

| Cost sector | Units | Value | Source |
| --- | --- | --- | --- |
| Time-related diagnostic procedures^‡^ |  |  |  |
| Team Assistant (Administration)^1^ | Hour | **26 €** | Collective agreement for Public Service (29) |
| Physician (Neurologist) ^2^ | Hour | **56 €** | Collective agreement of Physicians at university hospitals (30) |
| Psychologist^3^ | Hour | **40 €** | Collective agreement for Public Service (29) |
| Psychological Assistant^4^ | Hour | **29 €** | Collective agreement for Public Service (29) |
| Utilization of biomarkers^‡‡^ |  |  |  |
| Blood test^†^ | Quantity | **89 €** | German Hospital Federation (31) |
| Magnetic resonance imaging (MRI) | Quantity | **378 €** | German Hospital Federation (31) |
| Computer tomography (CT) | Quantity | **172 €** | German Hospital Federation (31) |
| Cerebrospinal fluid puncture (CSF) | Quantity | **118 €** | German Hospital Federation (31) |
| Fluorodeoxyglucose Positron emission tomography (PET) | Quantity | **644 €** | German Hospital Federation (31) |
| Florbetaben Positron emission tomography (PET) | Quantity | **1,917 €** | Self-reported cost data according to invoices |

^‡^ Cost for overhead (20%) and absent days due to holiday and sickness (16%) were included; ^‡‡^ Refer to full costs including material costs and cost of medical services; ^†^ Includes the following tests: hemoglobin, hematocrit, erythrocytes, leukocytes, thrombocytes, folic acid and / or vitamin B12, glutamate oxalacetate transaminase (GOT), aspartate aminotransferase (ASAT, AST), glutamate pyruvate transaminase (GPT), alanine aminotransferase (ALAT, ALT), gamma-glutamyltranspeptidase (gamma-glutamyltransferase (Gamma-GT), thyroid stimulating hormone (TSH), cholesterol, high-density-lipoprotein (HDL) cholesterol, low-density lipoprotein (LDL) cholesterol, creatine kinase (CK); ^1^Grade: “E6”/ “3 years of vocational training”; experience level “2” (in the 4^th^ year); ^2^ Grade: “Ä2”/ “medical specialist”; experience level “2” (in the 4^th^ year); ^3^ Grade: “E13”/ “University degree”; experience level “2” (in the 4^th^ year); ^4^ Grade: “E6”/ “3 years of vocational training”; experience level “2” (in the 4^th^ year).
