# Supplementary material for: Cost of diagnosing dementia in a German memory clinic
Source: Alzheimers Res Ther. 2017 Aug 22;9:65. doi: 10.1186/s13195-017-0290-6 (PMC5568303; doi:10.1186/s13195-017-0290-6)
Supplement: Supplementary file 3 — Table presenting a description of the time-related processes as well as the number and percentage of missing values that were imputed for each step in diagnosing dementia. ‡ Voice recording conducted by neurologist/psychiatrists. ‡‡ Written description of the voice recording. SD standard deviation (DOCX 14 kb) [file 13195_2017_290_MOESM3_ESM.docx]

**Supplementary Table 2:** Description of the time-related processes as well as the number and percentage of missing values that were imputed for each step in diagnosing dementia

|  | **Values** | | |  | **Missing values** | |
| --- | --- | --- | --- | --- | --- | --- |
| **Procedure** | **n** | **mean** | **SD** |  | **n** | **(%)** |
| Admission | 107 | 11.3 | 2.5 |  | 13 | (10.8%) |
| Anamnesis | 90 | 32.5 | 9.9 |  | 30 | (25.0%) |
| Physical examination | 88 | 10.9 | 3.2 |  | 32 | (26.6%) |
| Neuropsychological examination | 110 | 43.8 | 13.3 |  | 10 | (8.3%) |
| Test evaluation | 75 | 14.6 | 10.8 |  | 45 | (37.5%) |
| Instrumental diagnostic procedures | 120 | - | - |  | 0 | (0.0%) |
| Diagnosis conference | 112 | 6.6 | 2.0 |  | 8 | (6.6%) |
| Preparation physician letter^‡^ | 52 | 14.8 | 7.7 |  | 68 | (56.7%) |
| Writing physician letter^‡‡^ | 99 | 33.1 | 6.2 |  | 21 | (17.5%) |

^‡^ Voice recording conducted by neurologist/ psychiatrists; ^‡‡^ Written description of the voice recording; SD, standard deviation
